# The Relationship Between Nailfold Microcirculation and Retinal Microcirculation in Healthy Subjects

**DOI:** 10.3389/fphys.2020.00880

**Published:** 2020-07-24

**Authors:** Jiaxin Tian, Yuan Xie, Meng Li, Julius Oatts, Ying Han, Yiquan Yang, Yan Shi, Yunxiao Sun, Jinghong Sang, Kai Cao, Chen Xin, Labisi Siloka, Huaizhou Wang, Ningli Wang

**Affiliations:** ^1^Beijing Tongren Eye Center, Beijing Tongren Hospital, Beijing Institute of Ophthalmology, Capital Medical University, Beijing, China; ^2^Department of Ophthalmology, School of Medicine, University of California, San Francisco, San Francisco, CA, United States

**Keywords:** nailfold microcirculation, retinal microcirculation, nailfold capillaroscopy, optical coherence tomography angiography, relationship

## Abstract

**Objective:**

To evaluate whether the nailfold microcirculation is associated with retinal microcirculation in healthy subjects.

**Methods:**

Fifty subjects without systematic and ocular diseases were enrolled. Thickness of peripapillary retinal nerve fiber layer (RNFL), vessel density (VD) of radial peripapillary capillaries (RPCs), and superficial capillary VD in macular zone were measured with optical coherence tomography angiography (OCTA) in left eyes. Nailfold microcirculation, including capillary density, avascular zones, dilated capillaries, and hemorrhages was examined on the fourth digit of each subject’s non-dominant (left) hand with nailfold capillaroscopy (NFC).

**Results:**

After adjustment for relatively systemic factors, multivariate regression analyses showed a significant direct relationship between RNFL thickness and nailfold capillary density (OR = 1.09; *p* = 0.046). RNFL thickness and RPCs VD were negatively correlated with nailfold avascular zones (OR = 0.855; *p* = 0.007; OR = 0.596; *p* = 0.010). Superficial VD of parafovea was negatively associated with dilated nailfold capillaries (OR = 0.794; *p* = 0.012).

**Conclusion:**

In healthy subjects, nailfold capillary lower density and abnormalities are associated with reduced RNFL thickness and retinal VD. The results provide a theoretical foundation for relevant studies on ocular diseases with microvascular abnormalities and could contribute to pathogenesis understanding in the future. NFC and OCTA have the potential to identify risk factors and improve accuracy of the early diagnosis and treatment of ocular diseases, even systemic diseases with any microvascular component in clinical practice.

**Clinical Trail Registration:**

http://www.chictr.org.cn/index.aspx, identifier ChiCTR 1800017875.

## Introduction

Microcirculation is recognized as a crucial element in many diseases. Nailfold capillaroscopy (NFC), as a non-invasive, highly sensitive, and convenient technique, is designed to assess multiple features of the nailfold capillaries, including blood flow, density, and different kinds of abnormalities. It can provide visual inspection for microcirculation in living body. Since the 1970s, NFC has been used in many systemic disorder researches, like rheumatologic condition (Redisch et al., 1970), diabetes (Chazan et al., 1970), schizophrenia (Pokorny et al., 1970), and so on. Microvascular abnormalities are also recognized as important factors in ocular diseases, like retinal arterial occlusions, retinal vein occlusions glaucoma, non-arteritic anterior ischemic neuropathy, and glaucoma (Flammer et al., 2001). Recently, some studies demonstrated nailfold microcirculation abnormalities were encountered in ocular diseases involved in vascular factors (Pasquale et al., 2015; Chen et al., 2018; Philip et al., 2019). The development of optical coherence tomography angiography (OCTA) has revolutionized the resolution with which the retinal vasculature is visualized, allowing the potential to evaluate retinal microcirculation quantificationally. The previous literatures have showed there is reduction of retinal superficial vascular density measured by OCTA in some ocular diseases, even in systemic diseases (Vadalà et al., 2019). Based on these evidence, we hypothesized that nailfold capillaries, as the terminal capillary networks, were associated with retinal capillaries. To test this hypothesis, using NFC and OCTA, we conducted the first study to examine the relationship among nailfold capillaries, peripapillary retinal nerve fiber layer (RNFL) thickness, and the macular and peripapillary superficial vessel density (VD).

## Materials and Methods

### Study Participants

This was a cross-sectional study. All subjects were recruited from the general ophthalmology clinic at Beijing Tongren Hospital from September 2018 to June 2019. The study adhered the tenets of the declaration of Helsinki and was approved by the Ethics Committee of Beijing Tongren Hospital. All participants provided written informed consent after agreeing to participate. Inclusion criteria were: (1) healthy subjects over 18 and lower 65 years of age with no systemic or ocular health conditions; (2) no history of ocular trauma or surgery, no optic nerve or macular pathology; (3) best-corrected visual acuity better than or equal to log MAR 0.1; (4) intraocular pressure (IOP) less than or equal to 21 mmHg; and (5) refractive error between +1.0 and -6.0 diopters. Patients were excluded if any of the following criteria were met: (1) history of non-dominant hand trauma in 1 month; (2) currently menstruating women; (3) history of smoking; (4) history of alcohol abuse; (5) fever or general malaise; (6) hypertension, diabetes, hypertriglyceridemia, or any other systemic diseases; and (7) use of anticoagulant or antiplatelet medications.

Systemic variables collected for all recruited subjects included height, weight, heart rate, systolic blood pressure, and diastolic blood pressure (DBP). Systemic blood pressure was measured using an electronic sphygmomanometer (Omron, HEM-7211, Japan) in the seated position after at least 10 min of rest. A comprehensive ophthalmic examination, including slit-lamp biomicroscopy, best corrected visual acuity, IOP, and fundus examination, was performed by an experienced ophthalmologist. IOP was measured using a Goldmann applanation tonometer. Fundus imaging was performed using a non-mydriatic camera (KOWA non-myd α-DIII, Non-myd 7, JPN). Central corneal thickness and axial length were measured by Lenstar biometry (LS 900, Haag-Streit, United States). The vertical cup-to-disc ratio was acquired by spectral-domain OCT (RTVue-XR Avanti, Optovue, Inc., Fremont, CA, United States).

### OCTA and OCT Image Acquisition and Processing

**FIGURE 1:**
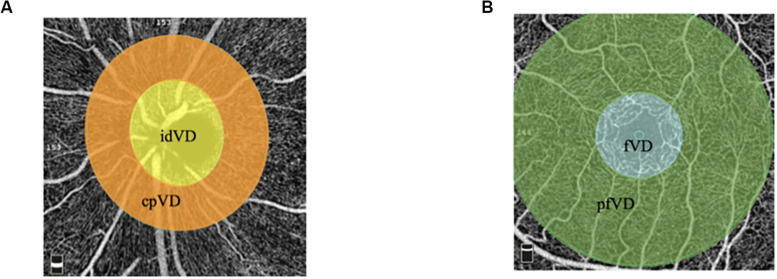
Retinal vessel density in different areas. **(A)** Vessel density map in radial peripapillary capillaries presented as wiVD. The yellow zone presented as idVD. The orange zone presented as cpVD. **(B)** Vessel density map in macular zone. The blue zone presented as fovea vessel density. The green zone presented as pfVD.

The OCTA imaging system (RTVue-XR Avanti, Optovue, Inc., Fremont, CA, United States) was performed on the left eyes of all subjects via undilated pupil and provides a non-invasive means for quantifying VD of radial peripapillary capillaries (RPCs) in optic disc zones and superficial vessels in macular zones. The A-scan rate is 70,000 scans/s with 840 × 10 nm scan beam wavelength. Structural en face angiogram showing VD with the percentage of the measured area occupied by vessels was captured. VD of RPCs was measured from the internal limiting membrane (ILM) to the posterior border of the RNFL using standard AngioVue software (version 2015.1.0.90). Results were obtained in three sections. Whole image VD (wiVD) was measured in the entire 4.5 × 4.5 mm field centered on optic disc; circumpapillary VD (cpVD) was calculated in the area defined as a 750-μm-wide elliptical annulus stretching from the optic disc boundary; inside disc VD (idVD) was inward the optic disc boundary. Superficial VD in the macular area was calculated from the ILM upper 3 μm to inner plexiform layer lower 15 μm. Foveal VD (fVD) was calculated in a 1 mm diameter of circle centered on the fovea of macula; parafoveal VD (pfVD) was measured in a 1 mm wide concentric annulus with an outer diameter of 3 mm (Yarmohammadi et al., 2017) (Figure 1).

The optic nerve head map protocol was used to obtain average peripapillary RNFL thickness by Avanti spectral-domain OCT system. The system contains 70-kHz axial line rate, 840-nm central wavelength, 22-mm focal spot diameter, and 5-mm axial resolution in tissue. RNFL thickness measurements were calculated in a 10 pixel-wide band along a 3.45 mm diameter circle centered on the optic disc.

Poor quality images were excluded, including: (1) a signal strength index of less than 47; (2) poor image formation; (3) irregular vessel patterns or disc boundary on the en face angiogram due to motion artifacts; (4) local deficiency or shadow induced by opacities or floaters; and (5) RNFL segmentation errors. If necessary, the location of the disc margin was manually adjusted for accuracy.

### Nailfold Capillaroscopy

**FIGURE 2:**
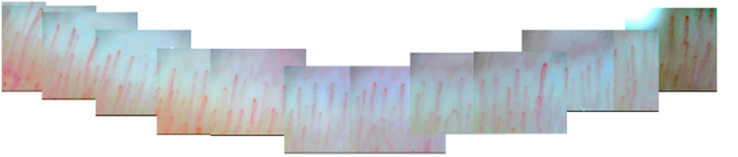
A splicing picture of panoramic nailfold capillaries from a healthy subject’s ring finger by nailfold capillaroscopy.

**FIGURE 3:**
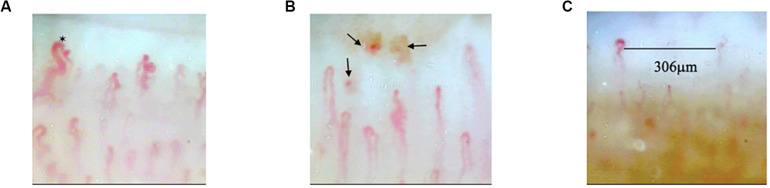
The images of abnormal nailfold capillaries. **(A)** Nailfold dilated capillaries (black hexagram). **(B)** Nailfold capillaries hemorrhages (black arrows). **(C)** Avascular zone in nailfold capillaries.

All participants were instructed to avoid strenuous activity or caffeine before examination. Examinations were performed between 9 and 11 o’clock to avoid possible diurnal variation. Subjects were arranged to have a rest at room temperature (20–25°C) for 15–20 min before measurement (Etehad et al., 2015). Given the most precise morphologic evaluation and reducing the risk of prior hand injury, NFC was performed on the forth finger in the non-dominant hand (Cutolo et al., 2003). All of subjects’ non-dominant hands were left hands. During the examination, the tested hand was kept at the same height as heart. The nailfold zone is the cuticle closest to the lunula. Cedar oil was applied to the nailfold to increase epidermal translucency. A microscope (JH-1004, Jiangsu Jiahua Electronic Instrument Co., Jiangsu, China) was used to capture the images of nailfold microcirculation at 280 magnification. More than 20 images were taken from each subject to exam panoramic nailfold capillaries (Figure 2). Image analysis was carried out to evaluate capillary density, number of avascular zones, dilated capillaries, and hemorrhages (software of Jiangsu Jiahua Electronic Instrument Co.) (Figure 3). Avascular zones were defined as the distance greater than 200 μm between two adjacent capillary loops in the proximal area. Dilated capillaries were defined as capillaries whose arterial limb diameter is larger than 15 μm or whose venous limb is larger than 20 μm (Etehad et al., 2015).

### Statistical Analysis

Mean values (standard deviation) were used to describe variables according with normal distributions. Medians (interquartile range) were used to describe variables with non-normal distribution. Categorical data were presented as counts and frequencies. Independent-samples *t*-tests, Mann–Whitney *U*-test, or Chi-square test was used to analyze the difference of systemic factors and retinal microcirculation for different kinds of variables, respectively, between subjects with and without each kind of nailfold abnormalities. We also compared systemic and retinal conditions between subjects with less nailfold capillary density (≤9/mm) and subjects with capillary density > 9/mm. Multivariable logistic regression analyses were used to test whether RNFL thickness and retinal VD were associated with different kinds of nailfold microcirculation conditions. A *P*-value < 0.05 was considered statistically significant. All statistical analyses were performed using commercial software (SPSS version 24.0; SPSS, Inc., Chicago, IL, United States).

## Results

**FIGURE 4:**
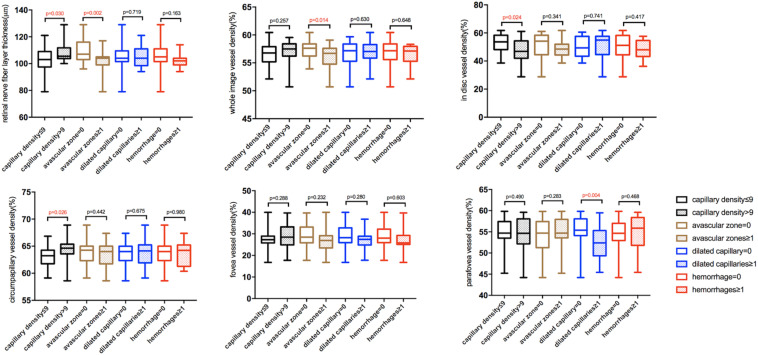
The box and whisker plots showing differences in retinal microcirculation of subjects with different nailfold microcirculation conditions. The five lines in each boxplot from the bottom to the top represent minimum, first quartile, median, third quartile, and maximum of the data, respectively.

Fifty eyes of 50 healthy subjects aged 18–64 years old were recruited in this study, including 23 males and 27 females (Table 1). Among all the subjects, 26(52%) had nailfold capillary density ≤ 9/mm; 23 (46%) had avascular zones; 17 (34%) had dilated capillaries; and nine (18%) had hemorrhages in the nailfold microcirculation. Compared with subjects whose nailfold density was > 9/mm, subjects with nailfold density ≤ 9/mm had thinner RNFL thickness (*p* = 0.030), less cpVD of RPCs (*p* = 0.026), but higher idVD (*p* = 0.024). Moreover, RNFL thickness and wiVD of RPCs were less in subjects with avascular zones than that in subjects without nailfold avascular zones (*p* = 0.002, *p* = 0.014). Finally, superficial pfVD was less in subjects with dilated capillaries than that in subjects without dilated capillaries (*p* = 0.004) (Table 2 and Figure 4).

**TABLE 1:** Demographic, systemic, and ocular characteristics of healthy subjects.

**Variables**	**Subjects, *n* = 50**
**Demographic characteristics**	
Age, median (IQR), years	27 (25, 31.25)
Sex, *n* (%)	
Male	23 (46%)
Female	27 (54%)
**Systemic characteristics**	
Heart rate, mean (SD), pm	76.80 (11.63)
Systolic BP, mean (SD), mmHg	116.02 (12.20)
Diastolic BP, mean (SD), mmHg	75.06 (8.06)
BMI, mean (SD), kg/m^2^	22.42 (3.28)
**Ocular characteristics**	
IOP, median (IQR), mmHg	15 (14, 16)
CCT, median (IQR), μm	548 (515, 573)
AXL, mean (SD), μm	24.40 (1.11)
VCDR, median (IQR)	0.39 (0.13)
RNFL thickness, median (IQR), μm	104.50 (100.75, 110.25)
**Radial peripapillary capillaries VD, %**	
Whole image VD, mean (SD)	56.80 (2.01)
Inside disc VD, mean (SD)	50.33 (7.57)
Circumpapillary VD, median (IQR)	64.02 (62.26, 65.00)
**Superficial capillary VD in macular zone, %**	
Fovea VD, median (IQR)	27.84 (25.74, 31.31)
Parafovea VD, median (IQR)	54.70 (52.87, 57.72)

*BP, blood pressure; BMI, body mass index; CCT, central corneal thickness; AXL, axial length; VCDR, vertical cup-to-disc ratio.*

**TABLE 2:** The differences of retinal microcirculation between subjects with and without one kind of nailfold capillary abnormalities.

**Parameters of retinal microcirculation**	**Capillary density**			**Avascular zones**			**Dilated capillaries**			**Hemorrhages**		
	**≤9, *N* = 24**	**9<, *N* = 26**	***P*-value**	**0, *N* = 27**	**1≤, *N* = 23**	***P*-value**	**0, *N* = 33**	**1≤, *N* = 17**	***P*-value**	**0, *N* = 41**	**1≤, *N* = 9**	***P*-value**
Avg RNFL thickness, μm	103 (97.50, 109.00)	105.50 (103.7, 111.75)	0.030^†^	109.37 (8.34)	101.87 (7.48)	0.002*	104.00 (101.50, 109.50)	104.00 (98.50, 111.00)	0.719^†^	106.73 (9.15)	102.22 (5.49)	0.163*
wiVD,%	56.46 (2.09)	57.11 (1.92)	0.257*	57.43 (1.55)	56.05 (2.25)	0.014*	56.70 (2.06)	56.99 (1.95)	0.630*	56.86 (2.03)	56.51 (2.02)	0.648*
idVD, %	52.81 (6.45)	48.03 (7.91)	0.024*	51.28 (8.54)	49.21 (6.24)	0.341*	50.07 (6.73)	50.83 (9.19)	0.741*	50.74 (7.73)	48.45 (6.87)	0.417*
cpVD, %	63.24 (61.75, 64.26)	64.62 (63.58, 65.37)	0.026^†^	64.26 (62.32, 65.00)	64.00 (61.74, 65.00)	0.442^†^	64.00 (62.35, 64.99)	64.13 (61.93, 65.25)	0.675^†^	64.01 (62.3, 65.00)	64.23 (61.2, 65.23)	0.980^†^
fVD, %	27.31 (25.8, 28.93)	28.47 (24.92,33.25)	0.288^†^	28.82 (5.12)	26.94 (5.56)	0.232*	28.59 (5.56)	26.81 (4.83)	0.280*	28.19 (5.25(	27.15 (5.98)	0.603*
pfVD, %	54.71 (3.44)	54.14 (4.44)	0.49*	53.86 (4.44)	55.11 (3.20)	0.283*	55.40 (54.11, 58.08)	52.38 (49.34, 55.29)	0.004^†^	54.66 (52.9, 57.02)	55.86 (51.8, 58.39)	0.468^†^

*t-test. ^†^Mann–Whitney *U*-test. ≤9, nailfold capillary density ≤ 9; 9<, 9 < nailfold capillary density; 0, no relevant nailfold abnormality; 1≤, existent relevant nailfold abnormalities.

Systemic factors, including gender, DBP, and age, were also associated with abnormalities of nailfold microcirculation (Table 3). Compared to subjects without avascular zones, subjects with avascular zones had significantly higher proportion of female (*p* = 0.042) and lower DBP (*p* = 0.029). Subjects with nailfold hemorrhages were older than subjects without hemorrhages (*p* = 0.032).

**TABLE 3:** The differences of demographic characteristics between subjects with and without one kind of nailfold capillary abnormalities.

3*Demographic characteristics	**Capillary density**			**Avascular zones**			**Dilated capillaries**			**Hemorrhages**		
	**≤9, *N* = 24**	**9<, *N* = 26**	***P*-value**	**0, *N* = 27**	**1≤, *N* = 23**	***P*-value**	**0, *N* = 33**	**1≤, *N* = 17**	***P*-value**	**0, *N* = 41**	**1≤, *N* = 9**	***P*-value**
Age, median (IQR), years	26(24, 29)	28(25.75, 33)	0.550^†^	27 (25, 32)	27 (24, 30)	0.696^†^	27(25, 31.5)	27(24.5, 31.5)	0.984^†^	26(24.5, 29.5)	32(27, 44)	0.032^†^
**Sex, *n***												
male	10	13	0.878^‡^	16	7	0.042^‡^	15	8	0.914^‡^	17	6	0.170^‡^
female	14	13		11	16		18	9		24	3	
**Systemic characteristics**												
Heart rate, mean (SD), pm	79.02 (10.73)	74.75 (12.26)	0.198*	77.63 (13.73)	75.83 (8.76)	0.577*	76.29 (10.93)	77.79 (13.18)	0.669*	78.20 (11.38)	70.44 (11.23)	0.070*
Systolic BP, mean (SD), mmHg	113.54 (12.71)	118.31 (11.48)	0.170*	118.48 (9.70)	113.13 (14.29)	0.123*	115.06 (12.71)	117.88 (11.28)	0.444*	115.20 (10.95)	119.78 (17.12)	0.312*
Diastolic BP, mean (SD), mmHg	74.25 (6.72)	75.81 (9.19)	0.500*	77.33 (7.65)	72.39 (7.86)	0.029*	74.12 (7.76)	76.29 (10.93)	0.255*	75.41 (7.85)	73.44 (9.28)	0.512*
BMI, mean (SD), Kg/m^2^	22.42 (3.43)	22.42 (3.21)	0.997*	22.17 (2.75)	22.71 (3.86)	0.567*	22.72 (3.21)	21.84 (3.44)	0.373*	22.20 (3.27)	23.42 (3.35)	0.320*

*t-test. ^†^Mann–Whitney *U*-test. ^‡^Chi-square test. ≤9, nailfold capillary density ≤ 9; 9<, 9 < nailfold capillary density; 0, no relevant nailfold abnormality; 1≤, existent relevant nailfold abnormalities. BP, blood pressure; BMI, body mass index.

**FIGURE 5:**
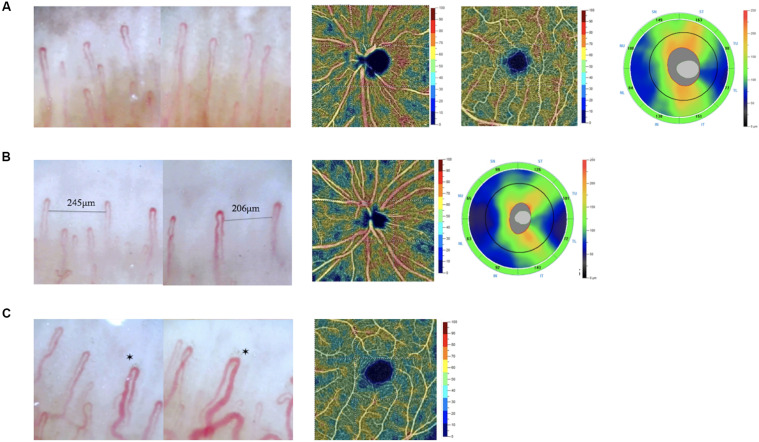
Images of nailfold microscopy, retinal VD maps, and RNFL thickness maps in different subjects. **(A)** A subject without avascular zone and dilated capillaries in nailfold. The whole image VD of the subjects was 57.84%. The parafovea VD was 59.35%. The average RNFL was 120 μm. **(B)** A subject with avascular zone in nailfold. The whole image VD of the subjects was 55.29%. The average RNFL was 95 μm. **(C)** A subject with dilated capillaries in nailfold. The parafovea VD was 51.25%. *dilated capillaries in nailfold.

We controlled for gender, DBP, and age to explore the relationship between nailfold microcirculation and retinal microcirculation. Multivariate regression analyses showed a direct relationship between RNFL thickness and nailfold capillary density (OR = 1.09, 95% CI = 1.001–1.187, *p* = 0.046). Thinner RNFL thickness and fewer wiVD were correlated with nailfold avascular zones (OR = 0.855, 95% CI = 0.763–0.957, *p* = 0.007; OR = 0.596, 95% CI = 0.402–0.883, *p* = 0.010). Fewer superficial pfVD was associated with nailfold dilated capillaries (OR = 0.794, 95% CI = 0.663–0.952, *p* = 0.012) (Table 4 and Figure 5).

**TABLE 4:** Multiple regression analyses among subjects with different kinds of nailfold microcirculation conditions in relation to the retinal microcirculation adjusted for age, sex, and DBP.

**Parameters of retinal microcirculation**	**Capillary density**		**Avascular zones**		**Dilated capillaries**		**Hemorrhages**	
	**OR(95%CI)**	***P*-values**	**OR(95%CI)**	***P*-values**	**OR(95%CI)**	***P*-values**	**OR(95%CI)**	***P*-values**
RNFL thickness	1.090 (1.001–1.187)	0.046	0.855 (0.763–0.957)	0.007	0.982 (0.914–1.055)	0.614	0.901 (0.797–1.018)	0.093
wiVD	1.193 (0.869–1.638)	0.275	0.596 (0.402–0.883)	0.010	1.025 (0.737–1.425)	0.884	0.989 (0.668–1.465)	0.955
idVD	0.924 (0.844–1.012)	0.088	0.884 (0.793–0.984)	0.024	1.045 (0.953–1.146)	0.346	0.982 (0.872–1.107)	0.770
cpVD	1.255 (0.928–1.696)	0.140	0.932 (0.689–1.260)	0.646	1.044 (0.774–1.408)	0.780	1.026 (0.704–1.494)	0.895
fVD	1.097 (0.957–1.258)	0.185	0.948 (0.827–1.088)	0.451	0.919 (0.800–1.055)	0.230	0.996 (0.850–1.166)	0.957
pfVD	0.951 (0.814–1.110)	0.525	1.100 (0.931–1.300)	0.261	0.794 (0.663–0.952)	0.012	1.058 (0.847–1.322)	0.617

## Discussion

In this study, it was the first time to analyze the difference in retinal microcirculation between subjects with and without one kind of nailfold capillary abnormality. The subjects with less nailfold density had thinner RNFL thickness and fewer cpVD, but possessed higher idVD. We supposed the RPCs in inside disc might compensate for less VD in circumpapillary. The subjects with nailfold avascular zones and dilated capillaries had thinner RNFL thickness and reduced retinal VD. The results are in line with previous study. Gugleta et al. used NFC to confirm subjects with or without vasospastic propensity, and then analyzed the difference in retinal vasodilation after flicker light. The results showed people with vasospastic propensity had lower maximum dilatory amplitude and missed augmentation of the maximum vasodilation, which suggested the diseases involved vasospasm not only in the affected organs, but also in the whole body (Gugleta et al., 2006). Gasser et al. testified, in glaucoma patients, the blood flow velocities in nailfold capillary were associated with the velocities ophthalmic artery (Gasser et al., 1999). For retinal vascular diameter, Branca et al. (2006) did not find the retinal arteriole or retinal venule diameters in subjects with vasospastic propensity were smaller than retinal vessels in subjects without vasospastic propensity. Kochkorov et al. (2006) got the similar results about retinal vascular diameter, whereas they further attested healthy vasospastic subjects had higher retinal spatial irregularity and an increased vessel diameter variation in heartbeat. Here, our results showed subjects with nailfold microcirculation abnormalities had lower retinal VD, further supported the relationship between peripheral and ocular circulation. We did not found any associations among idVD, fVD, and nailfold microcirculation. These two parameters could be lack of clinical significance with less practical application in previous studies. Besides, there was not significant association between retinal microcirculation and nailfold capillary hemorrhages. The reason could be limited sample size.

In analysis of systemic factors for nailfold microcirculation, we found age, gender, and DBP could be involved in nailfold abnormalities. Previous studies have showed nailfold capillary density increased with age of the subjects (Terreri et al., 1999; Hoerth et al., 2012). Here, even though the difference in age of subjects with different levels of naildfold capillary density was not significant, the Spearman rank correlation test showed age was positively correlated with nailfold capillary density (*r* = 0.324, *p* = 0.022). In this study, the proportion of hemorrhages of healthy subjects was within the range of other results (Pasquale et al., 2015; Chen et al., 2018). And, 46% of subjects had avascular zones, which was smaller when compared with the other result aimed at Chinese, 60.8% (Chen et al., 2018). The reason could be the subjects in this study were younger than the previous study. Besides, the avascular zone was encountered more often in women than in men and affected by DBP, which was in keeping with previous researches (Andrade et al., 1990; Ruaro et al., 2016). As to dilated capillaries, that we took a stricter definition, which could be a reason that the subjects with dilated capillaries in the present study were more than the results reported in the previous literature (Pasquale et al., 2015; Chen et al., 2018).

After the affecting factors controlled, thicker RNFL thickness was associated with higher nailfold capillary density, and the occurrence of nailfold avascular zones was associated with thinner RNFL and less dense RPCs. Similarly, the appearance of dilated nailfold capillaries was associated with a decline in retinal superficial capillary density. Previous studies also confirmed the relationship indirectly, which demonstrated ocular diseases accompanying with nailfold abnormalities as well as systemic diseases accompanying with retinal abnormalities. Pasquale et al. (2015) have performed NFC on 199 patients with POAG and 124 controls and found dilated capillaries, hemorrhages, and avascular zone were associated with POAG after eliminated age, gender, and other confounding elements. Philip et al. (2019) showed patients with exfoliation glaucoma and normal-tension glaucoma presented lower resting peripheral capillary blood flow when compared with subjects without glaucoma. The study by Chen et al. (2018) demonstrated patients with uveitis had higher tortuosity ratings, lower density, and dilated capillaries in nailfold. Vadalà et al. (2019) showed hypertensive patients with worse renal function had retinal thinning and less capillary density in OCTA. Previous studies also attested the relationship between systemic microcirculation and ocular diseases from other perspectives. Michalska and Ludmiła (2012) found significant rheological changes occurred in glaucoma, like enhanced erythrocyte aggregation and decreased erythrocyte deformability. The previous studies also showed elevated plasma endothelin-1 was associated retinal vein occlusions, anterior ischemic optic neuropathy, and glaucoma (Iannaccone et al., 1998; Flammer et al., 2001; Li et al., 2016). And the higher degree of carotid arterial stenosis contributed to the thinner RNFL thickness (Wang et al., 2017). However, at present, the study of nailfold microcirculation in ocular diseases is limited. Besides, the causal relationship between retinal vascular changes and ocular diseases has not yet been determined. Observing the occurrence and development of diseases from a systemic perspective helps us understand the pathogenesis of diseases. To our knowledge, this is the first time to directly link the manifestations of nailfold microcirculation with RNFL thickness and superficial retinal VD measured by OCTA, which reflect the structure and function of retina, together and further demonstrate the relationship between nailfold microcirculation and retinal microcirculation.

The mechanism of the relationship was still obscure. Anatomically, nailfold capillaries, macular capillaries, and RPCs are all terminal capillaries with commonalities including a single layer of endothelial cell vascular wall. Capillaries in the nailfold and retina are mainly regulated by vascular endotheliocytes and regulatory factors, not by autonomic nerve (Gugleta et al., 2006; Mozaffarieh et al., 2008). When there is minimal vascular autoregulation in these small vessels, hemorheological features play a more significant role in blood flow regulation. RPCs are the terminal arterioles following the path of the retinal nerve fiber (Henkind, 1967; Fouquet et al., 2017). There is also generally a strong correlation between RPCs density and RNFL thickness (Yu et al., 2014). Based on this, the relationship between nailfold microcirculation and RNFL thickness is receivable. For nailfold abnormalities, one of the possible contributing factors is serum matrix metalloproteinase (MMP)-9 levels, an essential substance involved in hydrolyzing extracellular matrix. Higher MMP-9 levels might also degrade the basement membrane of capillaries, deform the vessels, and lead to formation of avascular zone in nailfold capillaries (Lee et al., 2015; Pasquale et al., 2015). Circulating endothelial progenitor cells (EPCs) play a role in ischemia induced angiogenesis (Gurtner et al., 2008). Having fewer EPCs could affect the vascular genesis and contribute to avascular zone formation. It follows that a decreasing number of EPCs could also contribute to a decreased density of superficial retinal capillaries. Nailfold hemorrhages may be related to the presence of super activated platelets (SAPs), which accelerate platelet microparticles formation and create a prothrombotic environment (Morel et al., 2011), as well as decreased EPCs, which have anticoagulation properties (Hristov et al., 2003; Smadja et al., 2008). Besides, the function lessening of endothelium-dependent flow-mediated vasodilation could facilitate the occurrence of abnormal capillary dilation. Subjects with abnormalities in hemorheology, such as increased blood viscosity, erythrocyte aggregation, and slower flow velocity, could suffer more frequent appearance of dilated capillaries.

In this study, we connected nailfold microcirculation with retinal microcirculation in healthy subjects through two commonly used technologies, NFC and OCTA. Our findings are consistent with previous studies and further establish the clinical significance of the nailfold capillary study in ophthalmology investigation. There are some differences between nailfold capillaries and retinal capillaries, so we do not support to replace OCTA completely with NFC. The nailfold capillary is easily affected by external factors, like temperature, and more susceptible to trauma. On the contrary, retinal microcirculation could be affected by IOP, axial length, refractive error, and changes in the thickness and function of RNFL as well as ganglion cells (Yu et al., 2014; Shiihara et al., 2018; Chao et al., 2019). However, the OCTA is more objective. The NFC could provide more information about function of systemic microcirculation at dynamic state. There could be mutual assistance and complementation in these two examinations. Based on the results of this research and previous studies, we believe the nailfold condition could be a biomarker in ocular diseases involved in microcirculation abnormalities. The retinal microcirculation could be a biomarker in systemic microcirculation disorders.

Some limitations should be noted. The sample in the study was limited, a larger population study could provide a more powerful conclusion for relationship between retinal and nailfold microcirculation. As a cross-sectional study, our study did not provide any insight into changes in nailfold or retinal microvascular over time. More parameters, like nailfold capillary blood flow, morphology, and choroid thickness, could be analyzed for enhancing the perception of the relationship. Additionally, according to previous studies, we strictly controlled the influencing factors, like menstruation for women, smoking, alcohol consumption, to get a relatively clear association between nailfold and retinal microcirculation (Gugleta et al., 2006; Kochkorov et al., 2006). A study covering more confounding factors, which is to explore the relationship in subjects with different characteristics and influence, should be performed. Besides, when suffering the changes or stimuli in the external environment, whether the nailfold and retinal microcirculation will respond in a consistent or opposite manner is need to clarify.

From the above, the study showed, in healthy subjects, there was directly relationship between nailfold and retinal microcirculation. The results provide a theoretical foundation for the previous studies showing nailfold microcirculation in ocular diseases with microvascular abnormalities and testified the significance of the conclusions. Besides, at present, the application of NFC in ophthalmic research is limited. We hope the study could inspire more ideas and contribute to pathogenesis understanding in related diseases. Both NFC and OCTA have the potential to identify risk factors. Combined use of them may enhance the understanding of patient’s condition, improve accuracy of the early diagnosis, and contribute to therapy formulation in ocular, even systemic diseases with any microvascular component. A longitudinal study on relationship between nailfold and retinal microcirculation may provide more insight on microcirculation diseases.

## Data Availability Statement

All datasets presented in this study are included in the article/supplementary material.

## Ethics Statement

The studies involving human participants were reviewed and approved by the Ethics Committee of Beijing Tongren Hospital. The patients/participants provided their written informed consent to participate in this study.

## Author Contributions

JT, JS, HW, and NW contributed to study concept and design. JT, YX, YY, and YSu performed the study. JT and YSh drafted the manuscript. JT, KC, and YH performed statistical analysis. CX, YSh, YH, and JO revised the manuscript. NW contributed to administrative, technical, or material support and study supervision. All authors participated in and provided help to the study.

## Conflict of Interest

The authors declare that the research was conducted in the absence of any commercial or financial relationships that could be construed as a potential conflict of interest.
